# The impact of family function on post-traumatic reactions of Chinese adolescents infected with COVID-19: a latent profile study

**DOI:** 10.3389/fpubh.2023.1153820

**Published:** 2023-05-04

**Authors:** Mingtu Xu, Runhui Tian, Cong Fu, Jingyang Li, Dingyu Bi, Yan Wang

**Affiliations:** ^1^Department of Mental Health, First Affiliated Hospital of Jilin University, Changchun, China; ^2^Department of Politics and Education, Jilin Experimental Middle School, Changchun, China; ^3^Affiliated Middle School of Jilin University, Changchun, China

**Keywords:** COVID-19, post-traumatic reactions, family function, adolescents, latent profile study

## Abstract

**Background:**

Since the end of 2019, Corona Virus Disease 2019, also known as COVID-19, has broken out in various countries. However, the change of China's COVID-19 prevention and control policy and the sharp increase in the number of infected people are making the teenagers have post-traumatic reactions. Negative post-traumatic reactions include: post-traumatic stress disorder (PTSD), depression, anxiety. Positive post-traumatic reaction mainly refers to post-traumatic growth (PTG). The purpose of this study is to explore the post-traumatic reaction, which refers to PTSD, depression, anxiety and the co-occurrence pattern of growth after trauma and to further explore the influence of family function on different categories of Post-traumatic Reactions.

**Methods:**

Latent profile analysis (LPA) was used to explore the co-occurrence of PTSD, depression, anxiety, and PTG. Multiple logistics regression was used to analyze the influence of family function on different categories of post-traumatic response.

**Results:**

There were three categories of post-traumatic reactions in adolescents infected with COVID-19 adolescents infected with COVID-19, namely: growth class, struggling class, and pain class. Multivariate Logistic regression showed that the growth class and struggling class were affected by problem solving and behavior control in family function, while the growth class and pain class were affected by problem solving, roles, behavior control, and general functioning. Multiple logistic regression showed that the growth class and struggling class were affected by problem solving and roles.

**Conclusions:**

The findings of this study provide evidence for the identification of high-risk individuals and the provision of effective interventions in clinical practice, as well as the influence of family functioning on the different categories of PTSD among adolescents infected with COVID-19.

## Introduction

Since the end of 2019, the outbreak of novel coronavirus pneumonia (COVID-19) has occurred in various countries. The Omicron variant is still prevalent in many parts of China. As a public health emergency, COVID-19 not only causes trauma to the body of the infected person, but also has a certain impact on their psychology (1). At the end of 2022, with the change of China's COVID-19 prevention and control policy, the number of infected people may surge (2). Some studies have shown that individuals experience a number of post-traumatic reactions after quarantine due to COVID-19. Among them, post-traumatic stress disorder (PTSD) is considered to be the most typical and widespread negative psychological reaction. A psychogenic symptom caused by a traumatic event that is mainly manifested as: intrusion, avoidance, negative cognitive and emotional changes, and changes in arousal and reactivity. Recent studies have shown that 7.6% of adults have symptoms of PTSD after 10 days of isolation (3). Meanwhile, symptoms of depression and anxiety are also common negative psychological reactions associated with PTSD after experiencing a traumatic event. Studies have shown that the prevalence of anxiety is as high as 35.1% (4) and the prevalence of depression is as high as 20.1% (5) less than a month after COVID-19 pandemic. Under the background of COVID-19, the current research on the post-traumatic reaction of COVID-19 infected people also focuses on the relevant medical and health departments. In the research on the mental health status of COVID-19 infected people, there have been studies that have confirmed that a large number of COVID-19 patients have experienced mental health problems such as depression, anxiety and post-traumatic symptoms (6, 7), and the systematic evaluation has also found that the prevalence of depression symptoms (52%), anxiety symptoms (47%) and post-traumatic stress symptoms (26.9%) in patients infected with COVID-19 is high (8). For adolescents who crave interpersonal communication and have violent emotional fluctuations, psychological distress caused by COVID-19 are more serious. Previous studies have shown that: In a study of 8,079 adolescents in China, the prevalence of depression was as high as 43%, anxiety was as high as 37%, and combined symptoms of depression and anxiety were as high as 31%. Compared with adults, they were also more likely to suffer from PTSD and emotional problems (9, 10). During the COVID-19 pandemic, it was found that adolescents infected with COVID-19 exhibited a higher incidence of depression and anxiety (11), and adolescents infected with COVID-19 exhibited a higher risk of mental disorders, including anxiety, depression, post traumatic stress disorder, and sleep disorders (12).

In recent years, with the rise of positive psychology, the study of trauma is no longer limited to the negative results caused by traumatic events. In addition to negative psychological effects, individuals also experience positive psychological changes, the so-called post-traumatic growth (PTG). The post-traumatic growth scale compiled by Tesdeschi and other people, proposed a five-factor model of post-traumatic growth, namely, interpersonal relationship change, new possibilities, personal strength, spiritual change, and appreciation of life (13). Previous studies have shown that individuals develop PTG after experiencing COVID-19 pandemic (14). Qualitative research shows that many patients show obvious changes in different themes of post-traumatic growth such as coping strategies, existing growth, lessons learned from disease, new opportunities, and social growth after infection with COVID-19 (15). As a negative psychological reaction, PTSD, depression, anxiety and positive psychological results of PTG not only exist in the normal population under the background of COVID-19 (16, 17), but also exist in COVID-19 patients (18, 19).

Some studies have explored the relationship between PTG with positive psychological reactions and negative psychological reactions such as PTSD, depression and anxiety, but their relationship has been controversial. Some studies suggest that PTG is positively correlated with PTSD (20, 21), while other studies suggest that there is a negative correlation between the two (22). PTG is positively correlated with anxiety (23), and PTG is positively correlated with PTSD. In the studies of PTG and depression, PTG is negatively related to depression, but the degree is different (24). These controversial results suggest that different comorbidity patterns and severity of PTG may form different subgroups (25). Previous studies of PTG mainly evaluated individual scores on scales as a whole. However, the variable-centered approach fails to explore the heterogeneity among different individuals (26), while the emerging Latent profile analysis (LPA) can identify different categories of latent subgroups and analyze their differences (27).

Previous studies have applied this statistical approach to latent profile analysis of post-traumatic responses, such as: a latent profile study of PTG and PTSD in adolescent earthquake survivors 1 year after the Wenchuan earthquake identified three subgroups, namely, growth grade (high PTG, low PTSD), resilience grade (low PTG and PTSD), and symptom and growth grade (high PTSD and PTG). In the latent profile study of anxiety, three subgroups were also identified, namely: growth group, depression/anxiety/growth common group, and depression/anxiety group (28). The comorbidity patterns of PTSD, anxiety, COVID-19 related perceived threat, and courtesy stigma in adolescents have been explored in the context of post-traumatic reactions to COVID-19, which were divided into three subgroups: moderate PTSD group, mild comorbidity group, and severe comorbidity group (29). Among the positive psychological reactions of adolescents after experiencing the COVID-19, existing studies have also divided them into three subgroups, namely, limited positive changes, overall strong positive changes, and partial positive changes (30). Some studies have also conducted comprehensive discussions on the positive and negative psychological tendencies of individuals, such as fear of COVID-19 pandemic, depression, anxiety, stress, mindfulness, and resilience. The results of latent profile analysis can be divided into three groups, including high fear and moderate psychological symptoms group, low psychological symptoms and high mindfulness and resilience group, and high fear and high psychological symptoms and low mindfulness and resilience group (31). During the COVID-19 pandemic, the latent profile analysis of PTSD, depression and PTG in adolescents was divided into three groups: Growth group, Distress group and Struggling group (32). As far as we know, there is no research on the latent profile analysis of PTSD, depression, anxiety and PTG of adolescents infected after the outbreak of the COVID-19 epidemic to identify individual differences.

In the past few years of the COVID-19 pandemic, adolescents have to live with their caregivers, family conflicts generated in this process are very likely to affect the individual's psychological state (33). The family is an important place for the physical and mental development of adolescents. According to the family system theory, the family is composed of several subsystems, which are both interrelated and restricted to each other, so as to make the whole family operate effectively. Previous studies have shown that family function is an important predictor of individual PTSD, depression, and anxiety (34–36), and a favorable family environment and parental support can play a protective role in adolescent PTG (37). The risks posed by COVID-19 have a negative impact on family wellbeing. Favorable family relationships and family belief systems can provide family resilience, resist the risks posed by COVID-19, and provide a healing power in adversity (38). During the COVID-19 pandemic, many empirical studies explored the relationship between family function and individual post traumatic reactions. In the study of college students, favorable family function can alleviate anxiety among college students during the COVID-19 pandemic (39), while disorders of family function may affect adolescent PTSD. The higher the degree of adolescent family dysfunction, the higher the prevalence of PTSD (40). Longitudinal studies have also shown that favorable family relationships may alleviate psychological disorders among adolescents during the COVD-19 pandemic, family relationships are an important manifestation of family functions (41). In the group of patients infected with COVID-19, it shows that family support can reduce the psychological barriers caused by COVID-19 symptoms (42), and harmonious family relations can also promote the post-traumatic growth of COVID-19 infected people (43).

In the past, the research on the post-traumatic reaction of the post infection social population was relatively scarce for the ordinary people or COVID-19 infected people in medical units who went to hospital during the COVID-19 pandemic. In conclusion, this study proposed the following research objectives, the first of which was to identify the classes of PTSD, depression, anxiety and PTG of adolescents infected with covid-19 by latent profile analysis (LPA). The second goal is to explore the relationship between family function and classes of post traumatic reactions. This study hypothesized that the family function of adolescents infected with COVID-19 was related to different reaction classes after COVID-19 infection.

## Methods

### Participants

From December 10 to December 15, 2022, we contacted the school leaders of four middle schools in Changchun City, Jilin Province, China. We adopted a cluster random sampling method to select middle school students from four middle schools in Changchun City, Jilin Province for an online questionnaire survey. In each school, we randomly selected 2 to 4 classes at each grade. The students in the selected classes completed the survey using an online platform called Wenjuanxing in China. The survey link was sent to the student's mobile phone and the statement stating “I agree to participate voluntarily in the survey” was submitted to the participants before the survey. The students agreed to continue the survey. A total of 2000 middle school students infected with covid-19 volunteered to participate in the online survey. The inclusion criteria for COVID-19 infected adolescents were: individual confirmation (positive test) or possible COVID-19 infection (medical or self diagnosis), and the invalid questionnaire was eliminated. The invalid questionnaire included incomplete information provided, and all the question options were consistent with the questionnaire. Finally, 1,835 adolescents infected with COVID-19 provided an effective questionnaire. Among adolescents infected with COVID-19, the age is 14.05 ± 0.872 years old, including 992 boys, accounting for 50.2%, 913 girls, accounting for 49.8%, 1,392 only-children, accounting for 75.9%, 443 non-only-children, accounting for 24.1%, 128 single parent families, accounting for 7%, and 1,707 non-single parent families, accounting for 93%.

### Measures

#### Post-traumatic stress disorder

Post-traumatic Stress symptoms were investigated using the Post-traumatic Stress Disorder Checklist for DSM-5(PCL-5), a 20-item self-report scale. The 20-item scale corresponds to the 20 DSM-5 symptoms of PTSD, including intrusion, avoidance, negative changes in cognition and mood, and changes in arousal and reactivity. The scale is scored on a 5-point scale with 0 = indicating no distress, 1 = a little distress, 2 = moderate distress, and 3 = a great deal of distress. 4 = indicates extreme distress (44). The Chinese version of the scale has good reliability and validity (45). In order to better ensure that the post-traumatic stress symptoms we measured were caused by the traumatic event of the pandemic, the study explicitly asked the participants to return to the troubles caused by the pandemic in their lives, and the “traumatic event” in each item was replaced by “COVID-19”. The α coefficient of the scale in this study was 0.94.

#### Depression

Depressive symptoms were measured using the Patient Health Questionnaire-9 (PHQ-9), a self-rating Questionnaire for depression screening, with items written according to Diagnostic and Statistical Manual of Mental Disorders (DSM-IV) diagnostic criteria. The reliability and validity of the Chinese version of the PHQ-9 were good (46, 47). The scale consisted of nine four-category items, with none = 1, a few days = 2, more than half of the days = 3, and almost every day = 4. The Cronbach's alpha value of the scale in this study was 0.92.

#### Anxiety

Anxiety was assessed using the General Anxiety Disorder-7 (GAD-7) questionnaire. GAD-7 items were compiled according to the Diagnostic and Statistical Manual of Mental Disorders, Fourth Edition (DSM-IV) diagnostic criteria (48). The Chinese version of GAD-7 has good reliability and validity (49). Anxiety status in the past 2 weeks was assessed. The scale consists of seven four-category items that are assigned a score of 1point for none at all, 2 points for a few days, 3 points for more than half of the days, and 4 points for almost every day. The Cronbach's alpha value of the scale was 0.94.

#### Post-traumatic growth

Post-traumatic growth was investigated using the Post-Traumatic growth inventory (PTGI) developed by Tedeschi and Calhoun in 1996 (50). The scale consists of 21 items, which are divided into dimensions of interpersonal relationship, new possibilities, personal strength, and appreciation of life. The scale was scored on a 6-point scale from 0 to 5, and the higher the score, the higher the level of post-traumatic growth. The Chinese version of this study had good reliability and validity (51). The internal consistency reliability of the scale was 0.90 (Cronbach's alpha value).

#### Family function

Family function was assessed using McMaster Family assessment device (FAD), which was based on McMaster family function theory. According to the theory, the basic function of family is to provide certain environmental conditions for the healthy development of family members in terms of physiology, psychology and society (52). The Chinese version of the scale has good reliability and validity (53). The scale includes: problem solving, communication, roles, affective responsiveness, affective involvement and behavior control, and general functioning. The Cronbach's alpha value of problem solving was 0.66, communication was 0.72, roles was 0.75, affective responsiveness was 0.76, affective involvement was 0.67, behavior control was 0.73 and general functioning was 0.71. The scale is scored on a 4-point scale, where 1 is completely agree, 2 is agree, 3 is disagree, and 4 is completely disagree. The internal consistency coefficient (Cronbach's alpha value) of all items was 0.862.

### Statistical analysis

In this study, SPSS 21.0 was used to calculate the total Scores for each dimension of PTSD and PTG, as well as depression and anxiety, which were converted into Standardized z Scores, and Mplus 7.0 was used to perform latent profile analysis (LPA) on the calculated z scores (54). The fitting indexes include: AIC, Bayesian information criterion (BIC), sample size adjusted BIC (aBIC), Entropy, Lo Mendell Rubin likelihood-ratio test (LMRT), bootstrap likelihood-ratio test (BLRT), the lower the AIC, BIC, aBIC, the better the model fit. Entropy should be at least 0.8 and above, LMRT, BLRT need to achieve a significant level (*P* < 0.05), meanwhile, the proportion of individual profiles should not be < 5% (55). At the same time, considering the theoretical significance and simplicity of the model with the optimal profile, when the model with the optimal profile cannot be selected by the above-mentioned indexes, one can observe the changing values of different latent profiles AIC, BIC, ABIC and find inflection points to identify the model with the optimal profile (56–58). SPSS 21.0 was used to identify the relationship between family functioning and different categories using multi-nomial logistic regression.

## Results

### Post-traumatic reactions LPA results

The fitting statistics for latent profiles of the PTSD, PTG, depression anxiety are shown in Table 1. The results showed that LMRT and BLRT were significant when used in Class2, Class3, Class 4 and Class 5, demonstrating the heterogeneity of the post-traumatic reactions induced by covid-19 in the population of adolescents with infection. Entropy represents the classification accuracy, and the values of Entropy of Class 2, Class 3, and Class 4 and Class 5 are all >0.9, which belong to the high Entropy value, and the classification accuracy is high, among them, class 2 > class 3 > class 4 > class 5, but considering that AIC, BIC as well as aBIC during the decreasing process with increasing class, the trend of class 3 and class 4 is significantly slower. Meanwhile, the classification rate of Class 3 is 0.11–0.47, which is also simpler than the other classes. Therefore, Class 3 were selected as the optimal model in this study. Differences in indices of post-traumatic reactions among latent classes are in Table 2.

**Table 1:** Goodness-of-fit statistics for 2–6 class solutions.

**Class**	**AIC**	**BIC**	**aBIC**	**Entropy**	**LMR-LRT**	**BLRT**	**Proportion of categories (%)**
2	46,692.247	46,863.206	46,764.720	0.955	< 0.001^***^	< 0.001^***^	0.81/0.19
3	42,637.613	42,869.235	42,735.802	0.937	< 0.001^***^	< 0.001^***^	0.11/0.47/0.42
4	40,420.002	40,712.286	40,543.907	0.935	< 0.01^**^	< 0.001^***^	0.45/0.13/0.32/0.10
5	38,771.818	39,124.765	38,921.439	0.933	< 0.001^***^	< 0.001^***^	0.20/0.24/0.11/0.35/0.10
6	37,274.394	37,688.004	37,449.732	0.957	0.45	< 0.001^***^	0.07/0.31/0.09/0.07/0.43/0.12

AIC, Akaike Information Criterion; BIC, Bayesian Information Criterion; SS-BIC, Sample Size Adjusted BIC; LMR-LRT, Vuong-Lo-Mendell-Rubin Likelihood Ratio Test.

^***^p < 0.001.

**Table 2:** Differences standardized *z* scores for post-traumatic reactions among latent classes (*N* = 1,835).

**Class indices**	**Class 1 (*n =* 876) *M ±SD***	**Class 2 (*n =* 762) *M ±SD***	**Class 3 (*n =* 197) *M ±SD***	***F(p)***	***Post hoc* (Scheffé)**
In	−0.29 ± 0.47	−0.25 ± 0.54	2.30 ± 1.09	1,631.97^***^	1 < 3, 2 < 3
Av	−0.31 ± 3.34	−0.29 ± 0.34	2.52 ± 1.09	2,989.97^***^	1 < 3, 2 < 3
NA	−0.39 ± 0.49	0.01 ± 0.93	1.71 ± 1.10	583.90^***^	1 < 2 < 3
AAR	−0.34 ± 0.69	0.03 ± 0.99	1.42 ± 0.84	351.11^***^	1 < 2 < 3
De	−0.34 ± 0.75	0.14 ± 1.10	1.01 ± 1.08	191.61^***^	1 < 2 < 3
An	−0.31 ± 0.77	0.12 ± 1.03	0.93 ± 1.07	157.42^***^	1 < 2 < 3
PS	0.76 ± 0.54	−0.88 ± 0.66	−0.01 ± 0.77	1,420.68^***^	2 < 3 < 1
AL	0.69 ± 0.67	−0.83 ± 0.68	0.13 ± 0.76	999.85^***^	2 < 3 < 1
Re	0.75 ± 0.64	−0.87 ± 0.61	−0.01 ± 0.73	1,310.19^***^	2 < 3 < 1
NP	0.73 ± 0.69	−0.86 ± 0.57	0.09 ± 0.78	112.22^***^	2 < 3 < 1

In, Intrusions; Av, Avoidance; NA, Negative alterations in cognition and mood; AAR, Alterations in arousal and reactivity; De, Depress; An, Anxiety; PS, Personal strength; AL, Appreciation of life; Re, Relating to others; NP, New possibilities.

^***^p < 0.001.

**Figure 1:**
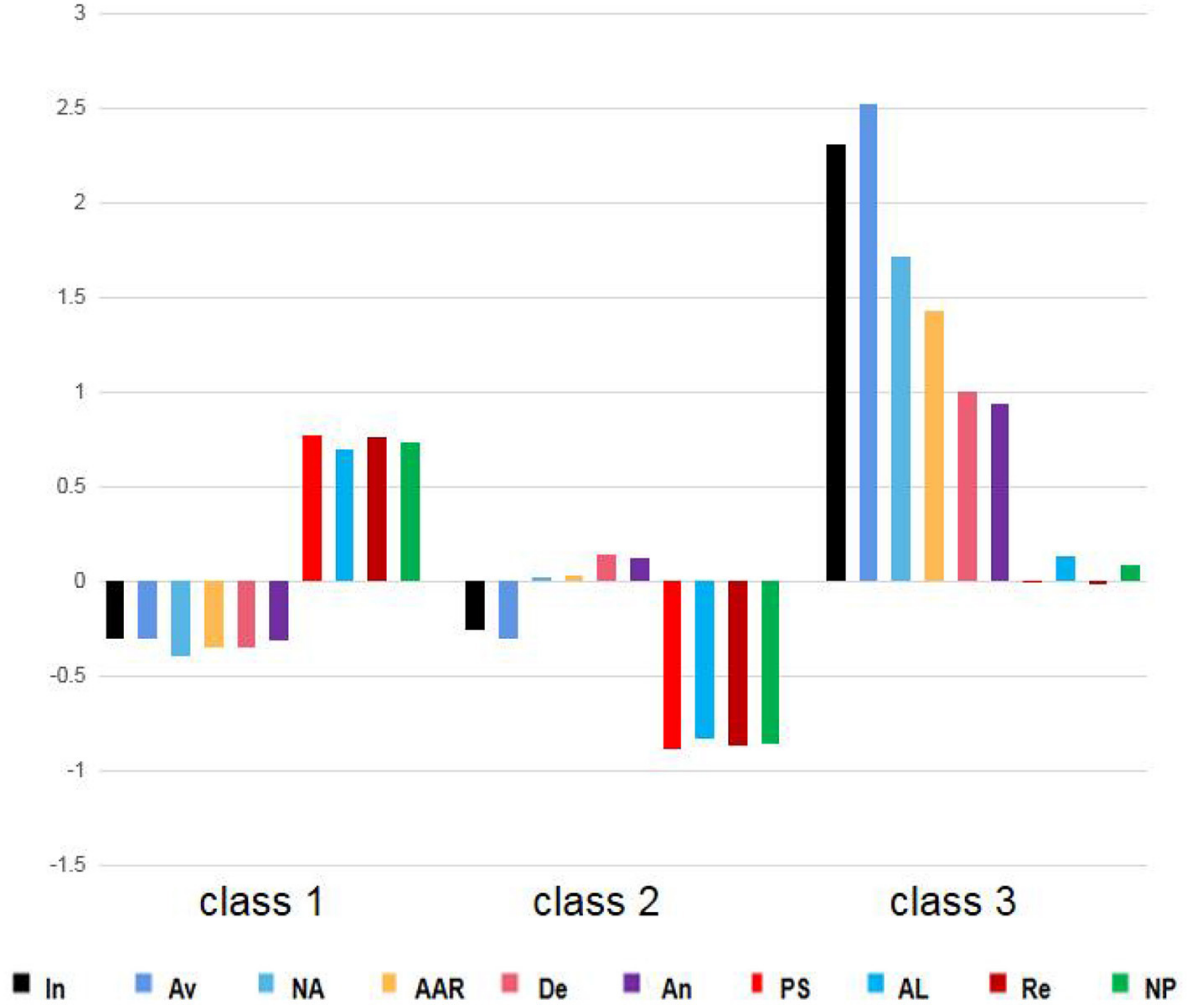
The three classes of post-traumatic reactions by latent profile analysis. In, Intrusions; Av, Avoidance; NA, Negative alterations in cognition and mood; AAR, Alterations in arousal and reactivity; De, Depress; An, Anxiety; PS, Personal strength; AL, Appreciation of life; Re, Relating to others; NP, New possibilities.

The distribution of PTSD, PTG, depression and anxiety profile model is shown in Figure 1. Class 1 shows that the class has relatively low PTSD, low depression and anxiety, and high PTG, so the class is named growth class, which accounts for 47% of the total. Class 2 shows that the class has moderate PTSD and depression and anxiety. And the low PTG, so the class was named as the struggling class, accounting for 42% of the total population. The Class 3 showed the high PTSD, depression, anxiety and moderate PTG, and the class was named as the pain class, accounting for 11% of the total population.

### Effects of family functioning on different classes of post-traumatic reactions

To understand the influence of family functioning on different classes of post-traumatic reactions, multiple logistic regression was used to test the predictive effects of different dimensions of family functioning on the formation of different classes of PTSD, depression, anxiety, and PTG. Among them, the growth class was used as the reference variable, and then the pain class was used as the reference variable to repeat the analysis. The results are shown in Table 3. Taking the growth class as the reference variable, in the comparison between the growth class and the struggling class, problem solving and behavioral control became the influencing factors. In the comparison between the growth class and the pain class, problem solving, roles, behavioral control, and general functioning became the influencing factors. Taking the struggling class as a reference variable, problem solving and roles became influential factors in the comparison process between the pain class and struggling class.

**Table 3:** Multiple logistic regression of family functions in differentiating distinct patterns.

	***B***	***SE***	***Exp(B)***	***95%CI***	***P***
**Growth class vs. struggling class**					
Problem solving	0.261	0.13	1.298	1.005–1.676	0.045^*^
Communication	0.118	0.166	1.125	0.812–1.559	0.479
Roles	0.228	0.195	1.256	0.857–1.841	0.242
Affective responsiveness	0.106	0.135	1.111	0.854–1.447	0.433
Affective involvement	0.044	0.136	0.957	0.733–1.249	0.747
Behavioral control	0.446	0.148	1.562	1.168–2.090	0.003^**^
General functioning	0.292	0.208	1.339	0.891–2.012	0.160
**Growth class vs. pain class**					
Problem solving	0.813	0.207	2.255	1.503–3.384	< 0.001^***^
Communication	0.045	0.284	0.956	0.548–1.668	0.874
Roles	1.075	0.334	2.931	1.523–5.642	0.001^**^
Affective responsiveness	0.158	0.234	0.854	0.540–1.351	0.500
Affective involvement	0.127	0.232	0.881	0.559–1.390	0.586
Behavioral control	0.57	0.259	1.767	1.064–2.935	0.028^*^
General functioning	0.921	0.347	2.513	1.272–4.965	0.008^**^
**Pain class vs. struggling class**					
Problem solving	0.552	0.199	0.576	0.390–0.851	0.006^**^
Communication	0.163	0.277	1.177	0.684–2.026	0.556
Roles	0.847	0.325	0.429	0.227–0.810	0.009^**^
Affective responsiveness	0.264	0.229	1.302	0.831–2.039	0.249
Affective involvement	0.083	0.226	1.086	0.697–1.693	0.715
Behavioral control	0.123	0.253	0.884	0.538–1.452	0.626
General functioning	0.630	0.338	0.533	0.275–1.033	0.062

^*^p < 0.05; ^**^p < 0.01; ^***^p < 0.001.

## Discussion

As we can see from our study, there are three potential categories of post-traumatic reactions from Chinese adolescents infected with COVID-19 consistent with previous results (32, 58), the growth class accounted for 47% of the total, the PTSD dimension, depression and anxiety scores were the lowest, the PTG dimension scores were the highest, and the struggling class accounted for 42% of the total, the PTSD dimension, depression and anxiety scores were in the middle. The scores of PTG were the lowest, and the pain class accounted for 11% of the total. The scores of PTSD, depression and anxiety were the lowest, and the scores of PTG were the middle. Consistent with previous research, a large number of adolescents fall into class 1, who have low PTSD, depression, and anxiety but high PTG scores, indicating that a considerable number of young people infected with COVID-19 have achieved positive psychological changes (59). Adolescents' cognitive ability is malleable, and it is easy to reshape their understanding of traumatic events when affected by traumatic events. A positive and optimistic view of traumatic events results in post-traumatic growth (60). It should be noted that compared with the growth class, the pain class had higher PTSD dimensions, depression, and anxiety scores than class 1, but lower post-traumatic growth than the growth class. Compared with the struggling class, the pain class had higher PTSD dimensions, depression, anxiety scores, and post-traumatic growth than the struggling class. This is different from previous studies that found high PTSD with high PTG class (58, 61) and high PTSD with low PTG class (32), and the reason may be that, differences in individual categories of post-traumatic reactions at different stages of the traumatic event.

After comparing growth class vs. struggling class, growth class vs. pain class, and pain class vs. struggling class, we found that problem solving was related to the post-traumatic reaction categories. In the process of solving problems, the family and its members grow up, the intimacy between family members is enhanced, the integrity of the family is maintained, and various functions of the family as a social unit are well played. The theory proposed seven dimensions to evaluate family function: problem solving, role playing, communication, emotional expression, involvement, control and values. Problem solving is the core dimension, and its process includes: identifying problems, thinking about various solutions to problems, selecting appropriate solutions and implementing them, and evaluating the effects of solutions. Other dimensions revolve around problem solving (62). Problem solving refers to the ability of the family to solve problems in order to keep the family functioning effectively. The problems faced by the family mainly originate from two aspects, material and emotional (63). If these two aspects can be dealt with effectively, then the family is a well-functioning family (64), in which the children can feel family support from their parents. In this way, the family can resist the negative effects such as PTSD, depression and anxiety caused by the COVID-19 pandemic (65, 66).

In the comparison between the growth class and the pain class, behavioral control was related to the post-traumatic reactions categories. According to the family function theory, behavioral control reflects the standardization of family members' behavior, which refers to the restriction of family members' behavior when a family responds to various environmental pressures, including risky behaviors, basic physiological needs and family members' communication (63). It has been noted that during the pandemic, adolescents and their families experienced changes in their normal lifestyles, which resulted in decreased mental health (67), while behavioral control allowed families to maintain a normal family routine during the pandemic, such as: Family habits such as bedtime and screen time require parental control of adolescents' behaviors, which may mediate the relationship between covid-19 related stress and family resilience and thus play a protective role in adolescents (68–70).

Roles in family function was found to be related to the post-traumatic reaction categories in a comparison of the struggling class with the pain class. Roles division refers to the relative position of family members in the family, the responsibility of family members, and the behavior pattern of family members repeatedly exercising when completing the family function. According to the family function theory, family functions are extensive, not only providing life for members, but also providing psychological support for members and meeting their developmental requirements (63). When completing the corresponding functions of the family, members should assume their corresponding roles, so as to maintain and manage the family system. In addition, in order to adapt to various emergencies, the family also has more requirements for the roles of each member. However, the chaotic division of family roles may lead to conflicts among family members, and lead to post traumatic stress disorder, depression, anxiety, and other negative emotions among adolescents (71–74).

General functioning was found to be related to the post-traumatic reactions categories in a comparison of growth class and the pain class. Mcmaster suggests that it is not any single factor that predicts good or bad family functioning, but rather that each factor can be used to make assumptions about the degree to which the family is effective in dealing with certain aspects of family life. A family may be unhealthy in some ways and functioning healthily in others. While general functioning in family functioning is an overall assessment of all dimensions of family functioning, dysfunctional family functioning, where the family is chaotic, rigid, and difficult to obtain the necessary resources to cope with stressful events (75), family functioning is an important protective factor for adolescents to cope with traumatic events (76).

This study explored the relationship between the various dimensions of family function and the categories of post-traumatic reactions in adolescents, but there are some limitations to this study. First, different age have different views on family function and different feelings about post-traumatic reactions (77, 78), and the changes in family function during adolescence are more prominent, such as: the requirement of individual independence, emotional changes, etc. Future research can explore the relationship between family function and post-traumatic reactions according to different age stages. Second, previous studies have analyzed the post-traumatic reactions in the process of traumatic events and after the occurrence of traumatic events, and the results of post-traumatic reactions are different, maybe the post-traumatic reactions is limited by the stage of the occurrence of the traumatic event. Therefore, longitudinal studies on the reactions at different stages of the traumatic event need to be further explored. Third, this study explored the relationship between adolescent family function and post-traumatic reactions experience post-COVID-19. Family function is dynamic and is affected by its development stage and specific life events, so the impact of family and individual previous experiences on post-traumatic reactions needs to be further explored (79, 80).

## Conclusion

This study found that there were three patterns of post-traumatic reactions in adolescents infected with COVID-19, namely, growth class, struggling class, and pain class. Multiple Logistic regression showed that the growth class and struggling class were affected by problem solving and behavior control in family function, while the growth class and pain class were affected by problem solving, roles, behavior control, and total function. The types of pain class and struggling class were affected by problem solving and roles.

## Data availability statement

The raw data supporting the conclusions of this article will be made available by the authors, without undue reservation.

## Ethics statement

The studies involving human participants were reviewed and approved by Ethics Committee of the First Hospital of Jilin University. Written informed consent to participate in this study was provided by the participants' legal guardian/next of kin.

## Author contributions

MX and RT drafted the manuscript and conceived and designed the study. RT and JL revised the manuscript. MX and CF drew the figures. DB and YW were responsible for the data acquisition. DB performed the data analysis. MX performed the statistical analyses. All authors have read and approved the final manuscript.
